# Publishing Research With Undergraduate Students via Replication Work: The Collaborative Replications and Education Project

**DOI:** 10.3389/fpsyg.2019.00247

**Published:** 2019-02-13

**Authors:** Jordan R. Wagge, Mark J. Brandt, Ljiljana B. Lazarevic, Nicole Legate, Cody Christopherson, Brady Wiggins, Jon E. Grahe

**Affiliations:** ^1^School of Psychology, Avila University, Kansas City, MO, United States; ^2^Department of Social Psychology, Tilburg University, Tilburg, Netherlands; ^3^Institute of Psychology, University of Belgrade, Belgrade, Serbia; ^4^Department of Psychology, Illinois Institute of Technology, Chicago, IL, United States; ^5^Department of Psychology, Southern Oregon University, Ashland, OR, United States; ^6^Department of Psychology, Brigham Young University-Idaho, Rexburg, ID, United States; ^7^Department of Psychology, Pacific Lutheran University, Tacoma, WA, United States

**Keywords:** replication, pedagogy, psychology, publishing, undergraduates, teaching, projects, open science

The Collaborative Replications and Education Project (CREP; http://osf.io/wfc6u) is a framework for undergraduate students to participate in the production of high-quality direct replications. Staffed by volunteers (including the seven authors^1^ of this paper) and incorporated into coursework, CREP helps produce high-quality data using existing resources and provides structure for research projects from conceptualization to dissemination. Most notably, student research generated through CREP make an impact: data from these projects are available for meta-analyses, some of which are published with student authors.

The call for direct replications of published psychological research has been pronounced and sustained in recent years (e.g., Lindsay, 2015), yet accomplishing this in light of the current incentive structure for faculty is challenging (Nosek et al., 2012). There is pressure for faculty to publish original research in high-impact journals and report significant effects (Franco et al., 2014), and so replication work often does not get the attention that it requires or deserves (Martin and Clarke, 2017). CREP harnesses the potential of student research to answer this call.

## CREP Background

CREP's primary purpose is educational: to teach students good scientific practices by performing direct replications of highly cited works in the field using open science methods. The focus on students is what sets CREP apart from other large-scale collaborations with similar methodological priorities, such as the ongoing Psych Science Accelerator (Moshontz et al., 2018), and one-off projects such as the Reproducibility Project: Psychology (Open Science Collaboration., 2015) and the Many Labs projects (Ebersole et al., 2016; Klein et al., 2018). The CREP approach also differs from typical undergraduate research projects because CREP results are aimed to have an impact on psychological science as a field.

To select the studies for crowdsourced direct replications, the CREP team samples the most highly cited papers from the top-cited journals in each of nine sub-disciplines published 3 years before the present year (e.g., 2010 in 2013, 2015 in 2018). From this sample, our administrative advisors (CREP student alumni) rate papers for how feasible^2^ they would be for a student to replicate in a semester, as well as how interesting students would find the topic. If there is more than one study in a paper, the CREP team selects just one for replication (typically the one judged as most feasible). The top-rated studies are then reviewed by one or more Executive Reviewers before making a final selection as a group. The CREP team then notifies the original authors of the study selections and requests materials and replication guidance with the goal of creating the most high-fidelity replication possible. Documentation of the study selection process can be found at osf.io/9kzje/.

**Figure 1:**
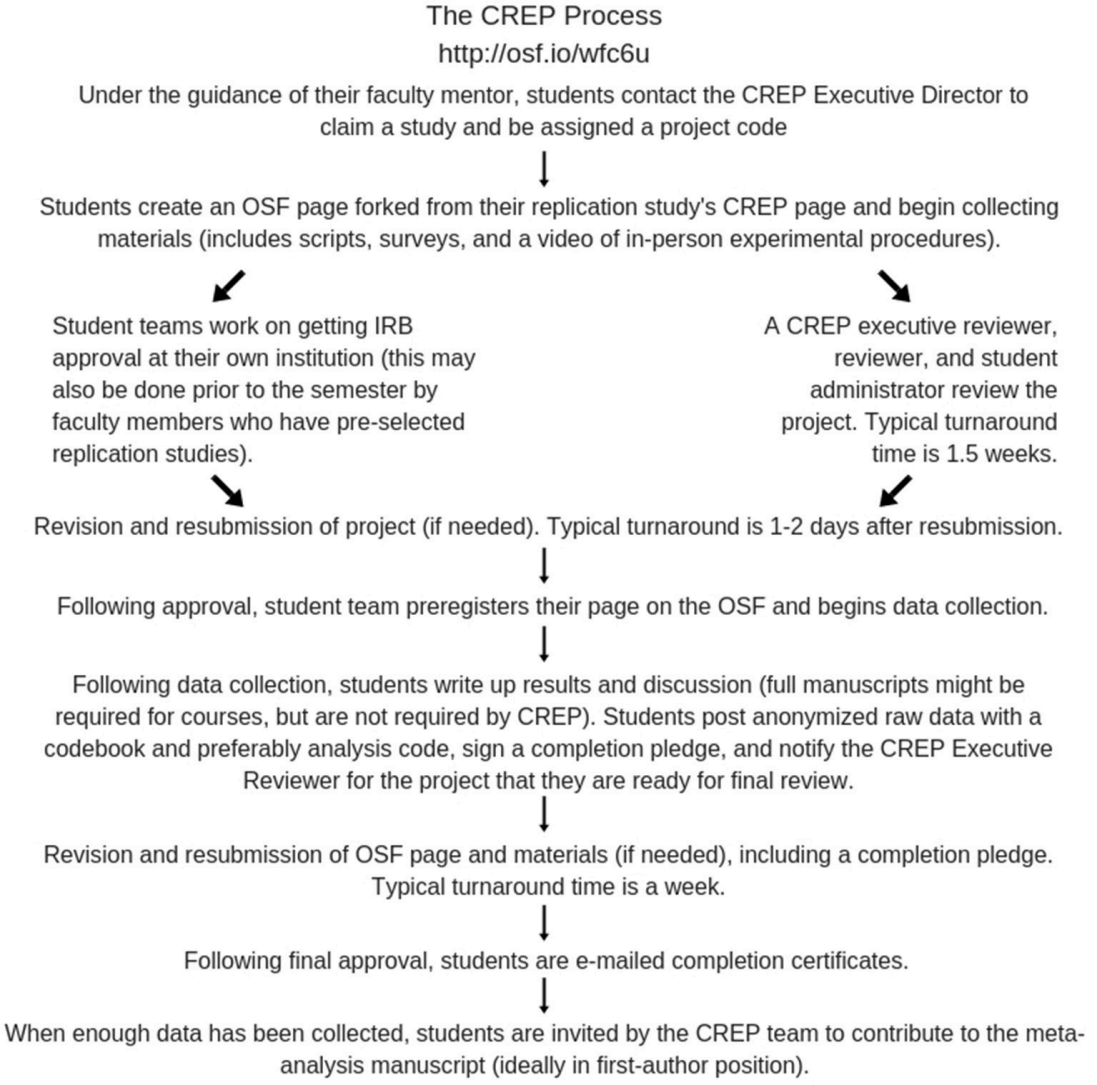
The CREP process for students.

For a student, the CREP process ideally looks like this: they are introduced to CREP by a faculty instructor at their home institution—typically in a research methods course, capstone course, or individual laboratory. Figure 1 shows the CREP process from that point on from the students' perspective. Student groups usually conduct direct replications, but can also include additional measures or conditions that the students add to test their own, original hypotheses. This Direct+ replication option can be performed out of student interest (e.g., theory-driven and based on previous findings) or out of a course or departmental requirement that students develop and test original hypotheses.

Figure 1 highlights that students are, along the way, participating in some of the critical requirements of open science and transparent methodology: open methods, open data, and preregistration of hypotheses (Kidwell et al., 2016). Students are also engaged in standard scholarly peer-review processes that many students do not get exposed to in their curricula. One notable piece of this process is that the CREP team participates in a revise-and-resubmit procedure of their project page until it meets the high standards the review team has set for replication fidelity and quality both before and after data collection. Being told about peer-review is one thing, but being a participant in the revise-and-resubmit process lends a greater appreciation for published scholarly work and how the peer review process works. For students who will enter academia, this training is essential for their careers. For students not pursuing academic careers, they gain skills in critically evaluating scientific claims by asking whether reported research has engaged these practices. For students who complete CREP projects and contribute to manuscripts, it prepares them for the revise-and-resubmit process that happens during the publication process.

## Dissemination of Student Work

CREP may be a more likely vehicle for student publication and citation compared to other teaching models that rely on student-generated hypotheses and single-site data collection. Student projects are rarely powered well enough for publication on their own. In a recent survey of instructors, who supervise research projects, we found that less than a third of instructors agreed with the statement that “Enough data is collected to make appropriate statistical conclusions” (only 4.9% strongly agreed) and less than a third of students complete a power analysis prior to data collection ((Wagge et al., manuscript in preparation). While close to ^2^/_3_ of instructors reported that the research questions for the projects were student-generated, only just over half agreed that student-generated hypotheses are interesting and <20% agreed that student research questions are typically grounded in theory. Unsurprisingly, these typical student projects completed as part of courses are not likely to lead to publication. Indeed, while instructors said that 79.5% of students presented their projects in class, just 30.4% reported presentations outside of class, and only 4.6% published in a journal. We believe these estimates may also be high given the nature of our specific sample (recruited from Twitter and Facebook methods groups, with large networks of open science advocates). For CREP replications, we anticipate that *all* completed student projects that meet our specifications will be included in meta-analyses. Indeed, this has been the case for our meta-analyses that have been published or are under review. The data are practically guaranteed life beyond the institution walls.

We strongly discourage contributors from writing their single studies for publication because any single CREP replication is not sufficiently powered to draw a strong inference. Instead, we wait until at least five samples are completed to begin a meta-analysis. Ultimately, the goal of the CREP is for completed projects to be reported in peer-reviewed manuscripts. There are currently several CREP meta-analyses in various stages of publication: two have been published (Leighton et al., 2018; Wagge et al., 2019), one has been submitted for publication (Ghelfi et al., 2018), one is in preparation (Lazarevic et al., manuscript in preparation), and an additional Phase 1 Registered Replication Report is in the review process (Hall et al., 2018) for a pilot partnership with the Psychological Science Accelerator (Moshontz et al., 2018).

Generally speaking, CREP can help students get first-hand experience with scientific dissemination in three ways. The first and most obvious way is that students can present their replication results at a conference (e.g., Peck et al., 2017). Second, students who complete replications that are used in CREP manuscripts have their OSF pages cited in those manuscripts. Students can therefore meaningfully contribute to science without needing the time and skill to write a professional paper themselves. OSF pages are also permanently and publicly available for other researchers to use. Our meta-analyses include only CREP direct replications, but other external meta-analyses may consist of conceptual replications and other, non-CREP direct replications. For example, a meta-analysis by Lehmann et al. (2018) of the red-rank-romance effect (e.g., Elliot et al., 2010) cites many of the individual projects completed by CREP groups. Therefore, by doing nothing beyond making their datasets publicly available (a requirement for CREP projects), students who completed replications for this project automatically gain cited authorship of their project's OSF page in a scholarly publication.

Third, and most importantly, students are invited to contribute to the authorship process when enough data has been collected for a meta-analysis. CREP has not been tracking student conference presentations systematically, but 27 CREP projects have been cited in three manuscripts currently published or under review, and 17 co-authors on these manuscripts were student CREP contributors. When possible, the CREP Executive team avoids taking lead authorship roles on meta-analysis manuscripts, offering these roles first to motivated students who have collected data and junior faculty who have supervised teams.

Replication work may be *more* likely to help students get published than other research models—while direct replications and null effect findings might not typically be considered “interesting” for journals, both null and confirmatory effects are interesting and important when they are replications of highly cited published works. For example, Royal Society Open Science recently committed to publishing close replications of work that was originally published in their journal (“Replication Studies”^3^ Further, the Psi Chi Journal has taken a step toward encouraging replications by offering authors a “Replication” badge in addition to the standard badges developed by the Center for Open Science (Kidwell et al., 2016). Recently, as a result, the first official CREP publication received the first “Replication” badge offered by any journal (Leighton et al., 2018). This publication included a student co-author and cited seven completed projects by students.

While we face many of the same challenges as other approaches to publishing with undergraduates (e.g., difficulty contacting former students to request their involvement), we believe that this approach is generally more productive than single-site projects as this has been the experience of several of us who have served as supervisors as well as manuscript authors. First, individual projects don't require collection from more participants than would be feasible for student teams in a typical semester. Second, students don't need a deep background in theory and the literature to run a CREP study and contribute to the manuscript. Third, publication doesn't require multiple studies or pretests, and we are unlikely to get feedback that more data needs to be collected to publish results.

## Benefits of CREP

Data from direct replications help establish credibility for the discipline. CREP also has the benefits for students and instructors. Students get training in cutting-edge research practices including pre-registration, open data, open materials, and large-scale collaboration. The selection of a replication study may lower barriers for beginning researchers, as students are not required to have extensive knowledge of a literature or research design before making a contribution with impact.

Instructors benefit from using CREP in four ways. First, CREP offers a supportive entry-point for faculty who are new to open science and large-scale collaborations. Second, because the data collected are meant to be included in a high-quality meta-analysis, CREP helps with fidelity and quality checks. Third, CREP eliminates the need for instructors to vet every hypothesis and design for student research projects. Instructors need not be experts in a topic to determine whether the hypothesis and design are relevant to the field and because we also try to provide stimuli and code for replications they do not need to learn new programs. Fourth, CREP is a rare opportunity for instructors to have a documentable experience blending teaching, scholarship, and close mentoring. These experiences are useful for tenure and promotion reviews. Faculty who choose who work as reviewers at CREP have an additional opportunity for meaningful international service experience.

In 5 years, more than 120 student groups have initiated CREP projects, and we hope to broaden the project's impact in future years. These projects offer the power, the rigor, and the fidelity needed for good replication work, all while providing the student the chance to learn, to publish, and to apprentice by following in the footsteps of scholars in the field. Given the CREP's benefits and initial success, we also believe this model can be successfully applied in other scientific disciplines.

## Author Contributions

JW wrote the original manuscript draft, revised the draft, and coordinated collaboration. MB, LL, NL, CC, BW, and JG provided feedback on drafts. All authors have made significant intellectual and time contributions to the CREP project.

### Conflict of Interest Statement

The authors declare that the research was conducted in the absence of any commercial or financial relationships that could be construed as a potential conflict of interest.

## Footnotes

^1^Jon Grahe is the Executive Director of CREP, Jordan Wagge is the Associate Director, and the other five authors are Executive Reviewers. The CREP was founded in 2013 by Jon Grahe, Mark Brandt, and Hans IJzerman.

^2^Feasibility considerations include sample size, sample characteristics, access to technology and equipment, and duration of study.

^3^Replication studies (n.d.). Retrieved from http://rsos.royalsocietypublishing.org/page/replication-studies).
